# Integration of HIV Care into Community Management of Acute Childhood Malnutrition Permits Good Outcomes: Retrospective Analysis of Three Years of a Programme in Lusaka

**DOI:** 10.1371/journal.pone.0149218

**Published:** 2016-03-04

**Authors:** Beatrice Amadi, Mercy Imikendu, Milika Sakala, Rosemary Banda, Paul Kelly

**Affiliations:** 1 Department of Paediatrics, University Teaching Hospital, Nationalist Road, Lusaka, Zambia; 2 Tropical Gastroenterology & Nutrition group, University of Zambia School of Medicine, Nationalist Road, Lusaka, Zambia; 3 Blizard Institute, Barts & The London School of Medicine, Queen Mary University of London, 4 Newark Street, London, United Kingdom; Asociacion Civil Impacta Salud y Educacion, PERU

## Abstract

**Background:**

While HIV has had a major impact on health care in southern Africa, there are few data on its impact on acute malnutrition in children in the community. We report an analysis of outcomes in a large programme of community management of acute malnutrition in the south of Lusaka.

**Programme Activities and Analysis:**

Over 3 years, 68,707 assessments for undernutrition were conducted house-to-house, and children with severe acute malnutrition (SAM) or moderate acute malnutrition (MAM) were enrolled into either Outpatient Therapeutic Programme (OTP) or Supplementary Feeding Programme (SFP) respectively. Case records were analysed using tabulation and unconditional logistic regression.

**Findings:**

1,859 children (889 boys, 970 girls; median age 16 months) with MAM (n = 664) or SAM (n = 1,195) were identified. Of 1,796 children whose parents consented to testing, 185 (10.3%) were HIV positive. Altogether 1,163 (62.6%) were discharged as recovered from acute malnutrition. Case fatality while in the programme was 4.2% in children with SAM and 0.5% in those with MAM (RR of SAM 10.9; 95%CI 3.4,34.8; *P*<0.0001), and higher in children with HIV infection (RR 5.2, 95%CI 2.9, 9.0; *P*<0.0001). In multivariate analysis, HIV (OR 5.2; 95%CI 2.6, 10.1; P<0.0001), MUAC <11.5cm (OR 4.1; 95%CI 2.2, 7.4; *P*<0.0001) and the first year of the programme (OR 1.9; 95%CI 1.0, 3.4; *P* = 0.04) all increased mortality. Children with HIV infection who were able to initiate antiretroviral therapy had lower mortality (RR 0.23; 95%CI 0.10, 0.57; *P* = 0.0008).

**Interpretation:**

Our programme suggests that a comprehensive community malnutrition programme, incorporating HIV care, can achieve low mortality even in a population heavily affected by HIV.

## Introduction

In many low and middle income countries, HIV and malnutrition frequently overlap. Many paediatricians and health workers in southern Africa, a region severely affected by both, recognize that HIV and malnutrition interact in a strongly negative way [1,2]. HIV affects many aspects of treatment of the malnourished child, including management of opportunistic infection and metabolic problems [3,4,5]. The case fatality among children with severe acute malnutrition (SAM) has historically been unacceptably high; a recent meta-analysis reported that mortality in SAM varies between 3 and 35% [6]. In HIV infected children with complicated SAM, inpatient mortality rates have been reported at 20% in Zambia [7], 10% in Bangladesh [8], 24% in Uganda [9] (but 44% if bacteraemic[10]), 19–21% in Kenya [11,12], and 42% in HIV-infected children in Malawi [13] (but up to 62% during long-term follow up [14]). Most of these deaths occur within 24–48 hours of admission in children brought late to hospital with severe illness (sepsis, diarrhoea, shock, pneumonia and tuberculosis) and those with underlying HIV infection [1,7].

Community Management of Acute Malnutrition (CMAM) enables the majority of children with acute malnutrition to be managed in the community [15–18]. The CMAM approach builds on evidence that protocol-based management reduces mortality [8]. CMAM is now the preferred approach as it ensures high coverage and better outcome: low case fatality rates (5% or less) are achievable when compared to exclusive inpatient care [19].

There are four components of CMAM [15]. First, community mobilization and participation, to ensure children with acute malnutrition are identified early at a community level before the onset of medical complications. Second, a Supplementary Feeding Programme (SFP) for management of children with moderate acute malnutrition (MAM). Third, an Outpatient Therapeutic Programme (OTP) for management of children with SAM with no medical complications and a good appetite. Children admitted into the OTP are put on Ready to Use Therapeutic Food (RUTF), which is made from peanut butter, milk, sugar, vegetable oils with added minerals and vitamins. Fourthly, Stabilization (inpatient) Care (SC) must be available for children with SAM with medical complications, poor appetite and those with generalised oedema.

However in Zambia, as in many other developing countries, only OTP and SC are being implemented widely while community mobilization is weak and SFP is not implemented at all, resulting in a large pool of children with MAM being left out. These “potential SAMs” only access care when they deteriorate to SAM. All too frequently, HIV care and CMAM operate independently. As a result, a majority of children being managed under CMAM who may be HIV infected are not identified and referred for Anti Retroviral Treatment (ART). Equally, most HIV infected children enrolled into ART programme who may have acute malnutrition are not referred for specific nutritional care in a CMAM programme.

The Department of Paediatrics at UTH set up a Community Nutrition Outreach Programme in mid-2009 in two Lusaka poor communities (Misisi and Kuku Compounds) the objective of which is early identification and care of undernourished HIV-infected and HIV-uninfected children. This programme aimed at addressing some gaps in the way CMAM is being implemented, including strengthening community identification of undernourished children alongside integration of HIV diagnosis and treatment. Here, we report some of the observational data collected in this Community Nutrition Outreach Programme.

## Description of Programme and Methods of Analysis

The ‘Misisi Community Nutrition Outreach Programme’ (MCNOP, which includes Kuku and Misisi) was initiated in May 2009 with a 5-month (May-September 2009) start-up phase, and we here report outcomes from October 2009-September 2012. A team of trained community health workers conducted house-to-house screening for undernutrition, and referred children to Misisi community nutrition outreach centre located within St Lawrence Catholic Parish based on the following criteria: MUAC <12.5cm, weight- for-age z score <-2, or bipedal oedema. They also referred any ill children who were not receiving medical treatment to the Outreach Centre for further evaluation by nurse counsellors and B Amadi. All the nurse counsellors are trained in Integrated Management of Childhood Illness (IMCI), CMAM, Child Growth Assessment based on WHO Growth Standards, Prevention of Mother to Child Transmission (PMTCT) of HIV and Infant and Young Child Feeding (IYCF).

At the Outreach Centre, nurse counsellors carried out a full anthropometric assessment (weight, height/length, MUAC) and checked for bipedal oedema. Children were classified as SAM (MUAC less than 11.5cm or bipedal oedema) or MAM (MUAC between 11.5cm and 12.5cm, and no bipedal oedema).

Children with SAM who had a good appetite were enrolled into OTP where they received RUTF, amoxicillin for 7 days, and single doses of vitamin A and mebendazole. These children were reviewed weekly and assessed using the IMCI algorithm including an appetite test: on review RUTF is given to the child by the caretaker under observation by nurse counsellor; consumption of one third of standard RUTF sachet or more is interpreted as “passing the appetite test”.

Indications for admission to the malnutrition ward at UTH for inpatient care included SAM with medical complications, generalised oedema (oedema 3+) and/or poor appetite. Once the medical complications were treated or were under control (e.g. started on Anti-Tuberculosis treatment), and appetite regained, children were discharged back to the Outreach Centre for enrolment into OTP and reviewed weekly.

Children with MAM were enrolled into SFP and reviewed every 2 weeks. They were given 3kg rations of High Energy Protein Supplement (HEPS), a corn-soya blend premixed with oil and sugar, mineral and multivitamin syrup and a single dose of mebendazole. Children with medical complications were referred for inpatient care and admitted to UTH, and upon discharge, such children were referred back to the Outreach Centre for enrolment into SFP.

Also enrolled into SFP were children discharged from OTP who were no longer severely malnourished (oedema resolved on 2 consecutive visits and with no severe wasting). All children were discharged from the programme once they no longer had signs of acute malnutrition (no longer MAM or SAM) and classified as ‘recovered’.

All caretakers of enrolled children participated in fortnightly cooking demonstrations to encourage optimum provision of a balanced diet at home.

### HIV testing

HIV testing was offered for all children enrolled in the programme after counselling of the caretakers; caretakers were also encouraged to be tested alongside their children. Refusal of the caretaker to be tested did not affect the child’s enrolment into the programme.

For children over 18 months of age, testing was by serology (Determine, Alere Medical Corporation, confirmed by Unigold, Trinity Biotech) only, and this test was used to classify children as infected or uninfected. Discrepant tests were resolved at UTH by way of a tie-breaker test. For children under 18 months, initial testing was by rapid test as above. Positive samples and negative samples from HIV-exposed children were tested by DNA-PCR on dried blood spots. Children in whom PCR detected viral DNA were classified as infected. Children under 18 months of age whose PCR was negative were classified as uninfected, as were children with seronegative mothers and negative rapid tests. If such children were still breastfeeding, repeat HIV testing was done as described above in accordance with National PMTCT guidelines.

All HIV infected children were referred to the Paediatric Centre of Excellence (PCOE) within UTH Department of Paediatrics or a clinic of the caretaker’s choice for enrolment into HIV care. Such children continued to attend the Outreach Centre as enrolled in either OTP or SFP for nutritional management and care.

### Data analysis

This was a retrospective analysis of a malnutrition screening and treatment programme in a very deprived community in southern Lusaka. Systematic, prospective data on death and follow-up were not available, so analysis was confined to known survivorship and death during inclusion in the SFP and OTP programmes until discharge. The primary outcome measures of interest were recovery, transfer to a less intensive part of the programme, and death. Potential risk factors for mortality, including the severity of malnutrition (MAM or SAM), were analysed separately and then included in logistic regression models. Definitions of outcomes were as described in *Community-based Therapeutic Care (CTC)*: *A Field Manual* [16]^.^ Potential risk factors for mortality were analysed separately and then included in unconditional logistic models; proportional hazards regression was not used as the date of death was often difficult to ascertain.

### Ethics

As this is a retrospective review of an outreach programme, it was granted exemption from ethical review by the University of Zambia Biomedical Research Ethics Committee on 4^th^ June, 2013. Informed consent for use of clinical records was not obtained in view of the retrospective nature of the review presented here and the scale of the program; the ethics waiver was granted on that basis. All patient information was recorded on paper files by the team of nurses and field workers, but all records were entered into a computer in an anonymised and de-identified fashion in accordance with the ethics waiver. The Nutrition Outreach Programme was not conducted for the purposes of research; no samples were collected for research; HIV testing was carried out purely for patient care to permit integration of HIV care into nutritional management which was the stated objective of the program.

## Results

Between October 2009 and September 2012, the study team carried out 68,707 screening examinations in the 2 communities, resulting in 1,859 children with acute malnutrition being enrolled into the Nutrition Outreach Programme (Table 1). Of these, 664 had MAM and 1,195 had SAM. Of 1,796 children with HIV status known, 185 (10.3%) were HIV infected. This proportion was higher (*P* = 0.02) in children with SAM (134 (12%) of 1,157) than in children with MAM (51 (8%) of 639). Altogether, 123 children were given ART during their period of treatment.

**Table 1:** Baseline characteristics.

	MAM (n = 664)	SAM (n = 1195)	*P*
Sex (M:F)	368:296	602:593	0.04
Age on recruitment (months, median, interquartile range IQR)	15 (10–21)	16 (11–22)	0.008
HIV infected	51/639 (8.0%)	134/1157 (11.6%)	0.02
WAZ^1^			
> -2	91 (14%	236 (20%)	<0.0001
< = -2	262 (40%)	302 (25%)	
< = -3	306 (46%)	655 (55%)	
WHZ^1^			
> -2	269 (43%)	517 (44%)	<0.0001
< = -2	351 (57%)	324 (27%)	
< = -3	0	341 (29%)	
HAZ			
> -2	160 (24%)	257 (21%)	<0.0001
< = -2	250 (38%)	343 (29%)	
< = -3	253 (38%)	596 (50%)	
MUAC (cm, median, IQR)	12.3 (12.0–12.5)	12.0 (11.4–13.0)	0.0002

^1^Children over 10 years of age not included in weight for age *z* score categories as WHO growth standard charts do not include these age groups; BMI-for-age is used as a measure of wasting. MUAC and weight for height are independent criteria for wasting; not all children who were wasted based on MUAC were necessarily wasted by the WHZ criterion [20]. Characteristics of children with MAM and SAM are described separately as these were important contributors to outcome, and the P values reported refer to the difference between MAM and SAM.

The overall cure rate improved over the 3 year period: 1st year 46.5%; 2^nd^ year 76.4%; 3^rd^ year 74.7%. The defaulter rate in the programme was high at 575 (30.9%); however, the defaulter rate reduced over the three year period as follows: first year 402 (48.4%); 2^nd^ year 100 (18.3%) and 73 (12.6%) by the 3^rd^ year. The families of 67 (3.6%) children were known to have relocated to communities outside the programme area.

### Outcomes in Moderate Acute Malnutrition (MAM)

**Fig 1:**
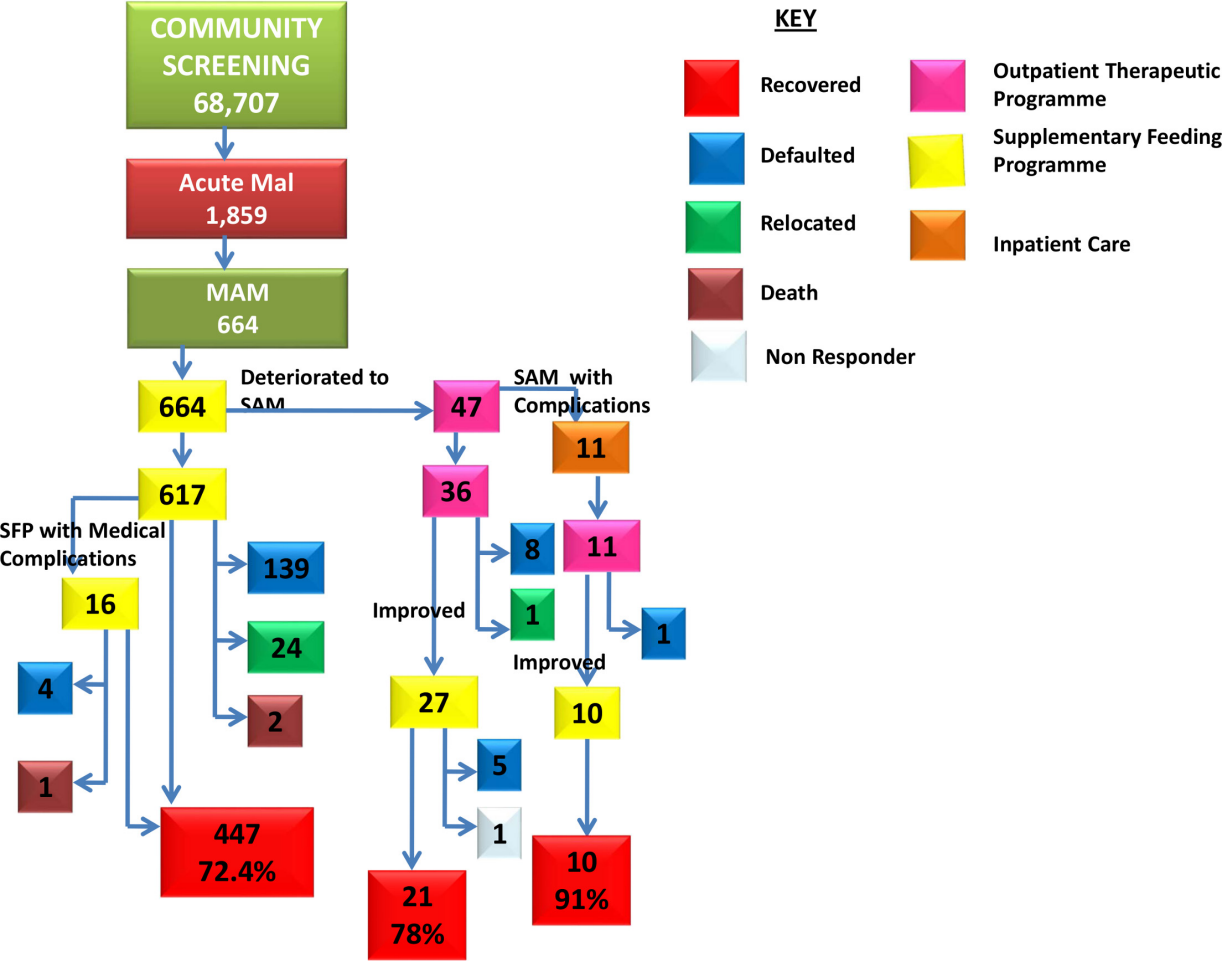
Flow of children with MAM through the programme. Children who were found to have moderate acute malnutrition (MAM) at screening were assessed, treated and followed up and their outcomes are shown. Some deteriorated to severe acute malnutrition (SAM) with or without complications. The colour key shows interventions and outcomes.

Over 3 years, 664 children (36% of the total 1,859 children with acute malnutrition) were enrolled into SFP, for a median duration of 9 weeks. Of these, 47 deteriorated to SAM, all of them having defaulted initially; they were enrolled into OTP then SFP as they improved from SAM to MAM. Overall, 478 (72%) children were discharged as recovered, 3 (0.5%) died, 157 (23.6%) defaulted while 25 (3.8%) relocated out of the programme area (Fig 1). Analysis by each programme year shows improved recovery rates and reduced defaulter rates (Table 2).

**Table 2:** Outcomes in children with MAM by year of programme.

Outcome	YEAR 1	YEAR 2	YEAR 3
Recovered	135(53%)	192(84.6%)	151 (83%)
Died	1 (0.4%)	2(0.9%)	0
Defaulted	113(44.5%)	26(11.5%)	18(9.8%)
Relocated	5 (2%)	7(3%)	13(7%)
Non Responder	0	0	1(0.5%)
**Total**	**254**	**227**	**183**

### Outcomes in Severe Acute Malnutrition (SAM)

**Fig 2:**
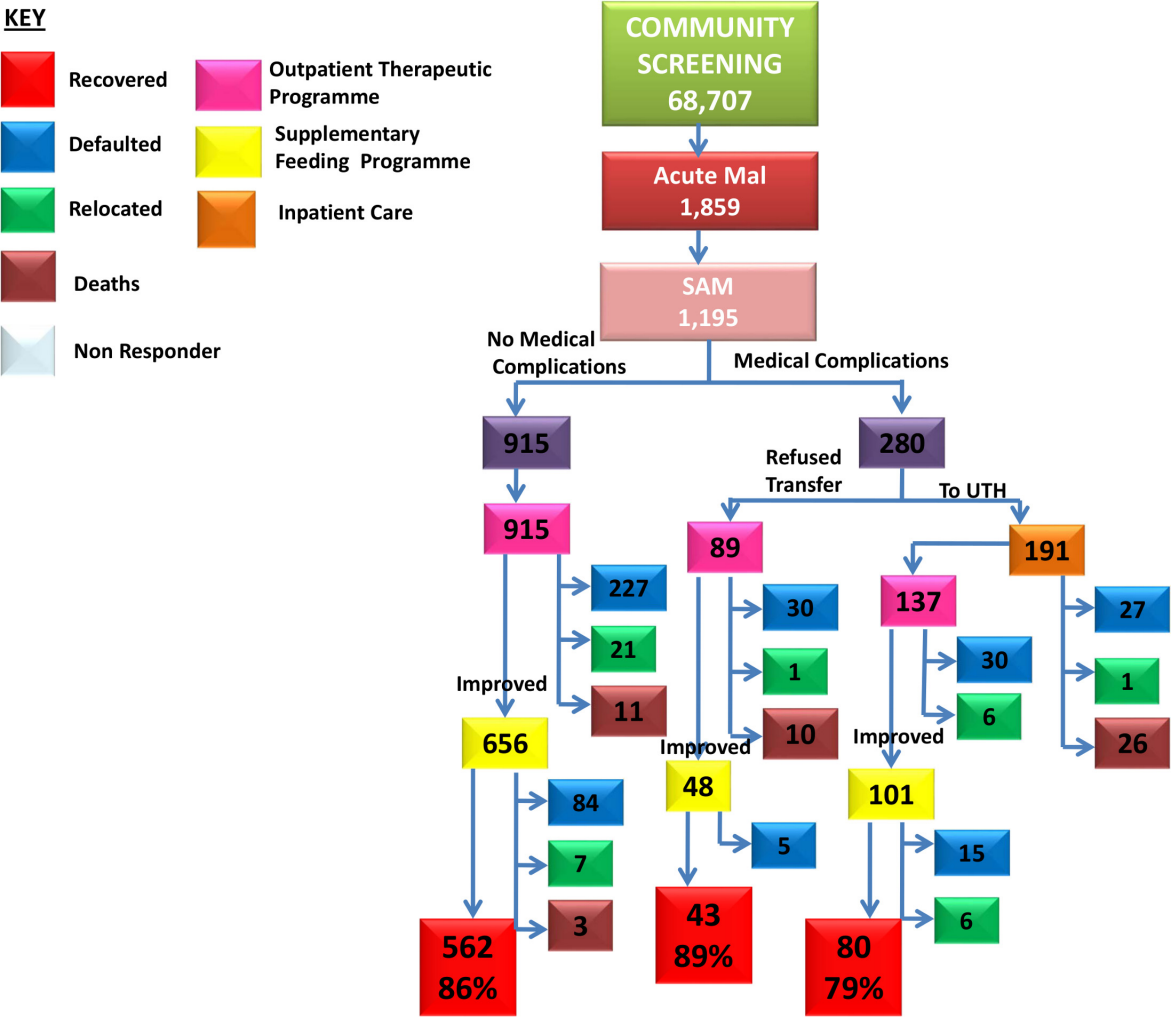
Flow of children with SAM through the programme. Children who were found to have severe acute malnutrition (SAM) at screening were assessed, treated and followed up and their outcomes are shown. Some improved to moderate acute malnutrition (MAM). The colour key shows interventions and outcomes.

A total of 1,195 children with SAM were enrolled into the outreach programme (Fig 2). They were classified into 2 groups as follows: 915 (77%) without medical complications were enrolled straight into OTP, and two hundred and eighty (23%) SAM with medical complications were referred for inpatient care. Caretakers of only 191 (68%) children were willing to be admitted to UTH; 89 (32%) declined. Again, outcomes improved after the first year of the programme (Table 3).

**Table 3:** Outcomes in children with SAM by year of programme.

Outcome	YEAR 1	YEAR 2	YEAR 3
Recovered	251 (43.5%)	225 (70.5%)	209 (69.9%)
Died	24(4.2%)	12(3.8%)	14(4.7%)
Defaulted	289(50%)	74(23.2%)	55(18%)
Relocated	13(2.3%)	8(2.5%)	21(7%)
Non Responder	0	0	0
**Total**	**577**	**319**	**299**

Of 915 children enrolled directly into OTP, 227 (25%) defaulted, 21 relocated, 11 (12%) died and 656 (72%) children improved nutritionally and were transferred into SFP. The 656 children who were transferred into SFP had good outcomes: only 3 (0.5%) died and 562 (86%) were discharged into the communities as recovered (Fig 2).

Of the 191 children admitted to UTH, 137 (72%) were successfully stabilized and discharged into OTP at the outreach centre; 26 (13.6%) died in UTH. There were no deaths recorded among the children in OTP. One hundred and one (101/137) children improved and were transferred into SFP. No deaths were recorded (Fig 2).

The outcome of those 89 (31.8%) children whose caretakers refused admission to UTH and were enrolled into OTP is of interest. Caretakers were advised to bring the children to outreach centres every 2 days for enhanced supervision. There were 10 (11.2%) deaths in this group while 48 (54%) improved and were transferred to SFP. The recovery rate in SFP was 43 (89%). These outcomes were not as good as in those who agreed to admission, justifying the current policy of admission for complicated SAM.

### Mortality

Overall, 53 (2.9%) of 1859 children in the programme died (S1 Table). Only 3 (0.5%) deaths occurred among children with MAM, and the great majority of the deaths, 50 (4.2%) were in the SAM group (RR of SAM for death 10.9; 95%CI 3.4,34.8; P<0.0001).

Of the 3 children with MAM who died, one child with medical complications (acute diarrhoeal disease) died in hospital while 2 children died at home while being followed up in SFP. The caretaker of one of these children refused transfer to UTH and the other child who was HIV infected but developed acute illness was not taken for medical treatment and died at home.

There were 50 deaths among children with SAM; 14 of these deaths occurred in children with no medical complications and were being followed up in OTP (11) and SFP after OTP (3). Seven of these children died in UTH while 6 died at home. All these deaths occurred during an outbreak of cholera and measles, but one death was from head injury. Twenty six children died in UTH during the stabilization phase while 10 deaths occurred in 89 children with medical complications whose caretakers refused transfer for inpatient care. Seven deaths occurred at home, while 3 died in UTH after being taken there by caretakers upon deterioration of illness. In univariate analysis, HIV positivity increased the risk of death (RR 5.2, 95%CI 2.9, 9.0; p = 0.000). In multivariate analysis, HIV (OR 5.2; 95%CI 2.6, 10.1; P<0.0001), MUAC <11.5cm (OR 4.1; 95%CI 2.2, 7.4; P<0.0001) and having been recruited in the first year of the programme (RR1.9; 95%CI 1.0, 3.4; P = 0.04) all increased risk of death during rehabilitation.

### HIV therapy

HIV infected children were referred to the HIV clinic at UTH for initiating ART, and 123 were started on it. Of 99 HIV infected children whose outcome is known, 7 were taking antiretroviral drugs at the time of enrolment. All of these children had recovered from acute malnutrition. Among the 125 children with HIV whose outcome was ascertained, 19 died, 6 of 83 while on ART (RR 0.23; 95%CI 0.10, 0.57; P = 0.0008).

## Discussion

In populations with a double burden of HIV and undernutrition, such as much of southern Africa, the interaction of these two drivers of childhood mortality is of prime importance. In response to the perception that such children need early diagnosis and integrated care, we set up a community-based programme, and here we report what we have learned. Our data indicate that HIV and severity of malnutrition contribute independently to mortality. However, implementation of this model of care in the community has led to very good outcomes, with a very satisfying 0.5% mortality in children with MAM, and a mortality of 4.2% in SAM despite a challenging burden of HIV infection. This provides strong support for the contention that ART can usefully be integrated into malnutrition programmes in the region. There is still considerable uncertainty surrounding the role of HIV testing and care in the management of SAM, and while some opinions and guidelines state that integration of HIV care is highly desirable [19,21,22], in a recent meta-analysis and Delphi consultation [6] no convergence was achieved on this issue. Nutritional interventions for children with HIV are known to be helpful [23], and the converse is self-evidently true. We believe, on the basis of the experience reported here, that malnutrition programmes in southern Africa which ignore HIV management can no longer be justified; HIV testing and referral must be integrated.

Our data, though obtained during the conduct of a large programme, have obvious limitations. Analysing mortality proved difficult given that this was not a research study but a retrospective analysis of a public health intervention programme. Ascertainment of outcome was very far from complete. However, we do know the reason for loss of follow-up in great majority of cases, which was moving house. This is often driven by economic factors, and we are not aware of processes which might lead to selective or biased ascertainment of death in HIV infected or uninfected children. The hazard ratio of HIV for mortality in our programme was 5.2 which is consistent with, but slightly higher than, hazard ratios for death of 2.0 in Malawi [24], 3.7 in Burkina Faso [25], or 4.0 in another study from Malawi [14]. Defaulter rates were generally high and mainly in HIV exposed and infected children, whose caretakers often fail to disclose their status to the spouse for fear of being divorced and may choose to drop out of the programme. However, as indicated above, the rates reduced to acceptable levels (the SPHERE standard [26]) by the 3^rd^ year of the programme. Success in reducing the defaulter rate was due to a dedicated team of field workers and nurse counsellors who carried out defaulter tracing every afternoon. This included focus group discussions with community members on the importance of completing nutritional rehabilitation together with messages on the need to test for HIV and enrolment into care programmes of all infected people. Additionally, a drama group was contracted to give messages through sketches on various health topics, including nutrition, HIV/AIDS prevention and treatment, hand washing, home chlorination of drinking water, management of diarrhoea at home, childhood immunization, and use of bed nets for prevention of malaria.

The interpretation of the apparent protective effect of anti-retroviral therapy is much more difficult. It may be that deteriorating health contributed to inability to initiate ART which might make failure of ART initiation merely a marker of impending death. It is now a matter of high priority to conduct controlled trials to resolve the issue when to introduce ART for children in malnutrition programmes.

One of the lynchpins of the CMAM concept is that there should be movement between SC, OTP and SFP, yet in Zambia the SFP is not comprehensively implemented. This is essential component of CMAM in our experience. Without an effective SFP a high proportion of MAM children will deteriorate to SAM, and we know that mortality is related to severity: early enrolment into therapy is the crux of the challenge of achieving low mortality rates. Mortality in our MAM group was very low at 0.5%. Furthermore, an effective SFP is critical for caring for children with SAM who are recovering [15,17,18.19]. Our readmission rate in this programme was low at 5%, and in our view this is largely attributable to having an effective SFP.

Are these findings generalisable? Zambia has a high burden of HIV infection but we do not know if these findings would be reproduced in other southern African countries. Further work is needed in this region. Our findings therefore cannot mandate a change of strategy, but there are several reasons to encourage integration of HIV care into CMAM in heavily affected countries. First, hospital-based studies consistently demonstrate that HIV increases mortality in childhood malnutrition [14,25]. Second, screening for malnutrition offers an excellent opportunity to screen for HIV in heavily affected communities, and these children then need treatment. Third, the profile of infectious disease found in malnourished children in many ways resembles that found in HIV/AIDS (bacteraemia, diarrhoea, tuberculosis), and experience in diagnosing and treating these infections is very valuable. Fourth, HIV infection frequently presents with malnutrition in children, so children with MAM and SAM in southern Africa will have a higher proportion of HIV infected children than children without malnutrition. Hospital-based paediatricians have appreciated for many years that treating severe, complicated cases is very difficult and that early identification of nutritional disorders is highly desirable. While community-based screening may seem like a resource-intensive approach, the results seem to justify it. Whereas hospital-based treatment of complicated SAM carries high mortality in our experience, the same team and same approaches appear to have been rewarded with better results by identifying and treating malnourished children earlier, in the community.

## Supporting Information

S1 Table Additional information on children who died. (PDF)
