# Supplementary material for: Integration of HIV Care into Community Management of Acute Childhood Malnutrition Permits Good Outcomes: Retrospective Analysis of Three Years of a Programme in Lusaka
Source: PLoS One. 2016 Mar 4;11(3):e0149218. doi: 10.1371/journal.pone.0149218 (PMC4778761; doi:10.1371/journal.pone.0149218)
Supplement: S1 Table — (PDF) [file pone.0149218.s001.pdf]

S1 Table Additional information on children who died

	Year Enrolled	Month Enrolled	Month/Year Died	HIV Status	On ART	Probable Cause of Death	Place Died	Additional observations
**SAM WITH COMPLICATIONS AND TRANSFER TO UTH**								
1	2010	January	Jan, 2010	Neg		SAM	UTH	
2	2010	March	June, 2010	Neg		Measles	UTH	
3	2010	March	May, 2010	Pos	Yes	SAM	UTH	
4	2010	April	Apr, 2010	Neg		SAM	UTH	
5	2010	April	May, 2010	Neg		SAM	UTH	
6	2010	April	May, 2010	Neg		SAM	UTH	
7	2010	April	May, 2010	Pos	Yes	SAM	UTH	
8	2010	May	May, 2010	Pos	No	SAM	UTH	
9	2010	May	Jun, 2010	Neg		SAM	UTH	
10	2010	June	Sept, 2010	Neg		Domestic violence	UTH	
11	2010	July	Aug, 2010	Neg		SAM	UTH	
12	2010	July	Oct, 2010	Pos	No	SAM	UTH	
13	2010	August	Sept, 2010	Pos	Yes	SAM	UTH	
14	2010	October	Nov, 2010	Neg		SAM	UTH	
15	2010	October	Nov, 2010	Neg		SAM	UTH	

16	2010	November	Nov, 2010	Pos	No	SAM	UTH	
17	2011	February	Feb, 2011	Pos	Yes	SAM	UTH	
18	2011	Septembe r	Nov, 2011	Neg		SAM	UTH	
19	2011	October	Nov, 2011	Neg		SAM	UTH	
20	2011	November	Mar, 2012	Neg		SAM	UTH	
21	2011	December	Dec, 2011	Neg		SAM	UTH	
22	2011	December	Jan, 2012	Neg		SAM	UTH	
23	2012	January	Jan, 2012	Neg		SAM	UTH	
24	2012	January	Mar, 2012	Neg		SAM	UTH	
25	2012	January	Mar, 2012	Pos	Yes	SAM	UTH	
26	2012	May	May, 2012	Pos	No	SAM	UTH	

SAM WITH COMPLICATIONS AND REFUSED TRANSFER TO UTH

27	2009	October	February, 2010	Pos	No	SAM	UTH	
28	2009	November	November, 2009	Neg		SAM	Home	
29	2010	May	June, 2010	Pos	No	SAM	Home	
30	2010	October	November, 2010	Neg		SAM	UTH	
31	2010	October	December, 2010	Pos	No	SAM	UTH	
32	2010	December	January, 2011	Pos	No	SAM	Home	
33	2011	April	January, 2012	Pos	No	SAM	Home	Mother observed selling RUTF

34	2011	October	November, 2011	Neg		SAM	Home	
35	2011	November	January, 2012	Neg		SAM	Home	
36	2012	May	May, 2012	Neg		SAM	Home	

SAM WITHOUT COMPLICATIONS

37	2009	November	March, 2010	Neg		Head Injury	UTH	
38	2010	January	March, 2010	Neg		Cholera-like illness	UTH	
39	2010	March	June, 2010	Neg		Measles	UTH	
40	2010	March	May, 2010	Pos	Yes	SAM	UTH	
41	2010	May	June, 2010	Neg		SAM	Home	
42	2010	June	July, 2010	Neg		SAM	Home	
43	2010	September	October, 2010	Pos	No	SAM	Home	
44	2010	September	October, 2010	Declined		SAM	Home	
45	2010	November	May, 2011	Pos	Yes	SAM	UTH	
46	2011	January	February, 2011	Neg		SAM	UTH	
47	2011	September	December, 2011	Neg		SAM	UTH	Diarrhoea, vomiting
48	2012	February	May, 2012	Neg		SAM	UTH	
49	2012	May	May, 2012	Neg		SAM	Home	
50	2012	June	September, 2012	Neg		SAM	Home	

MAM

51	2009	November	November, 2010	Neg		Diarrhoea	Home	
52	2010	December	February,	Neg		Diarrhoea &	UTH	

			2011			vomiting		
53	2011	July	August, 2011	Pos	No	Not clear	UTH	

SAM, severe acute malnutrition; MAM, moderate acute malnutrition; UTH, University Teaching Hospital; RUTF, ready-to-use therapeutic food
